# Personalizing prostate cancer diagnosis with multivariate risk prediction tools: how should prostate MRI be incorporated?

**DOI:** 10.1007/s00345-019-02899-0

**Published:** 2019-08-09

**Authors:** Ivo G. Schoots, Anwar R. Padhani

**Affiliations:** 1 Department of Radiology and Nuclear Medicine, Erasmus University Medical Center, P.O. Box 2040, ’s-Gravendijkwal 230, 3000 CA Rotterdam, The Netherlands; 2 Paul Strickland Scanner Centre, Mount Vernon Cancer Centre, Northwood, UK

**Keywords:** Prostate cancer, Biopsy, Magnetic resonance imaging (MRI), Risk stratification, Multivariate risk prediction, Risk calculator, Nomogram

## Abstract

Risk-based patient selection for systematic biopsy in prostate cancer diagnosis has been adopted in daily clinical practice, either by clinical judgment and PSA testing, or using multivariate risk prediction tools. The use of multivariable risk prediction tools can significantly reduce unnecessary systematic biopsies, without compromising the detection of clinically significant disease. Increasingly multi-parametric magnetic resonance imaging (MRI) is performed, not only in men with a persistent suspicion of prostate cancer after prior negative systematic biopsy, but also at initial screening before the first biopsy. The combination of MRI and multivariate risk prediction tools could potentially enhance prostate cancer diagnosis using multivariate MRI incorporated risk-based models to decide on the need for prostate MRI, but also using MRI results to adjusted risk-based models, and to guide MRI-directed biopsies. In this review, we discuss the diagnostic work-up for clinically significant prostate cancer, where the combination of MRI and multivariate risk prediction tools is integrated, and how together they can contribute to personalized diagnosis.

## Introduction

Magnetic resonance imaging (MRI) is an increasingly useful tool for clinically significant prostate cancer detection and has recently come to the forefront in the diagnostic work-up in many countries [1–3]. Its utility has been demonstrated by multiple prospective studies including randomized clinical trials [4, 5] and multiple, systematic analyses that consistently show improvements in biopsy avoidance, reductions in the detection of indolent disease and improved detection of clinically significant disease [6, 7]. Prospective multicenter studies as the recently published ‘MRI-first’ [8] and ‘4 M’ trials [9] show the beneficial effects in prostate cancer detection in biopsy-naïve men, when MRI is combined with systematic and targeted biopsies.

However, these studies do not directly address the question on who benefits from the MRI approach. As for systematic biopsy, this is likely to be biopsy-naïve men with elevated serum PSA levels and/or abnormal digital rectal examinations, and those who are deemed to be at persistent elevated risk of harboring significant cancers despite prior negative or non-explanatory systematic biopsies. However, clear guidance cannot be derived from the available literature, because inclusion criteria for all MRI studies have been restricted to those with higher risks, and studies evaluating the benefits of MRI according to clinical risk factors are few [10, 11]. The 2019 EAU prostate cancer guideline recommends not limiting biopsies to pre-specified PSA thresholds, as there are many other factors (e.g., symptoms, age, race, family history, PSA kinetics, and digital rectal examination findings) that also inform on the decision to biopsy [1].

To aid decision-making with regard to the need for biopsy, multivariate risk-based prediction tools have been developed to assess the likelihood of having clinically significant prostate cancer [12]. These risk calculators stratify men for further biopsy testing or clinical monitoring using readily available clinical parameters. Multivariate risk prediction tools can also be used to decide on the need for prostate MRI by enriching patient selection on one hand and avoiding MRI testing on the other [13, 14]. Thereafter, adding anatomical and functional information after MRI to multivariate risk-calculator assessments refines the underlying risk of clinically significant cancer and thereby modulates the need for biopsy, also informing on biopsy methods so as to increase biopsy yields, by identifying targets that are likely to harbor clinically significant disease [15, 16].

In this review, we conceptualize the diagnostic work-up of patients suspected of having clinically significant prostate cancer using combinations of MRI and multivariate prediction tools and explore how they together can enable individualized risk-adapted diagnostic strategies.

## Risk assessments

Risk assessments for prostate cancer diagnosis aim to identify disease-related factors that have the potential to cause disease or increase the risk of developing disease. Factors that enhance disease likelihood include older age, family predisposition, African ethnicity, elevated PSA serum levels (or derivatives), abnormal rectal examination, and genetic factors. These risks require weighting for the target condition, which is preferably clinically significant prostate cancer. To integrate multiple risk factors, multivariate risk-based prediction tools (i.e., risk calculators or nomograms) have been developed. Risk calculators provide numerical outputs that indicate higher likelihood of prostate cancer (therefore needing biopsy), and, in contradistinction, identify which men do not need further testing, so avoiding further testing.

This individualized risk-adapted strategy for prostate cancer detection enables the balancing of benefits versus harms [17, 18]. It is important to identify only those men that are likely to benefit from timely diagnoses. Benefit is, therefore, the detection and appropriate treatment of clinically significant prostate cancer. The harms include redundant (unnecessary) testing and the likelihood of having complications of testing (such as rectal bleeding, urine retention, bacteremia, and urosepsis). Remembering also that many of the cancers detected may never become clinically evident, thereby leading to over-diagnoses and over-treatments [17], thus contributing to harms.

**Fig. 1:**
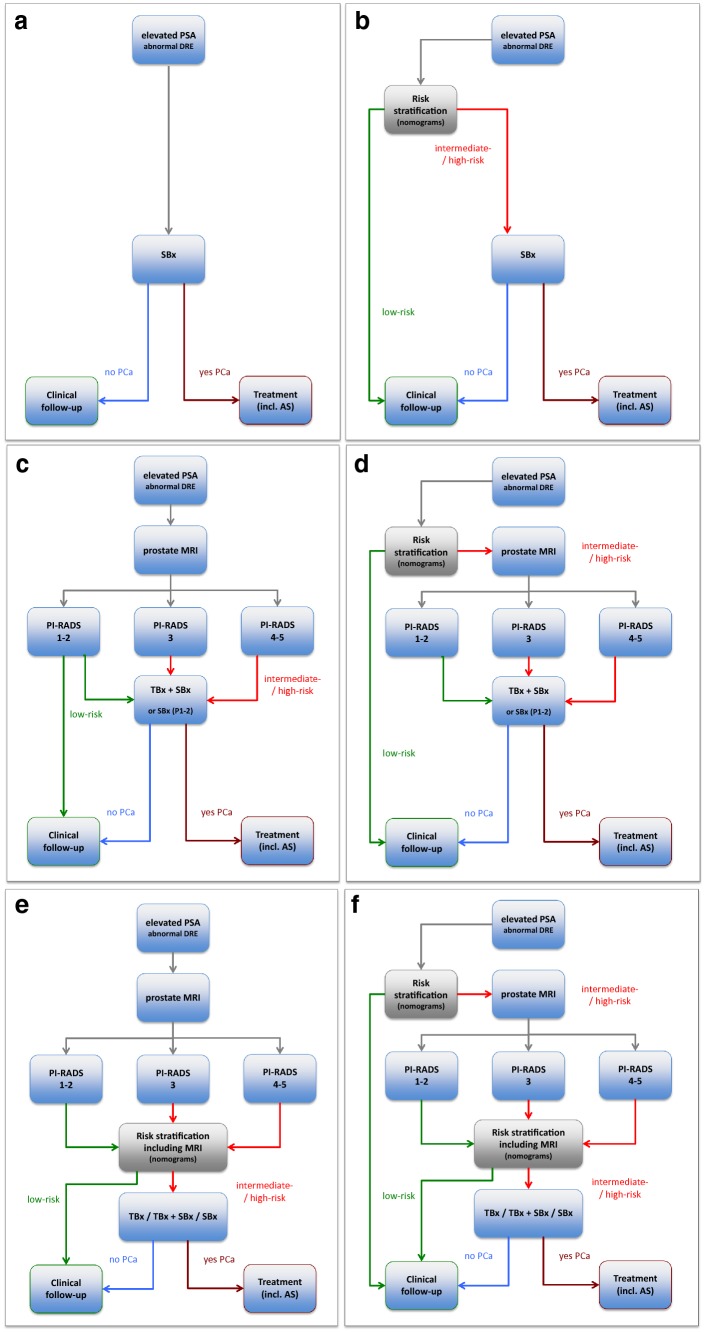
Diagnostic flowcharts of men with elevated prostate-specific antigen (PSA) and/or abnormal digital rectal examination (DRE), with combinations of risk stratification w/o prostate MRI. Risk assessment with **a** PSA only, **b** multivariate risk prediction tools only, **c** prostate MRI only, and **d** multivariate risk prediction tools: when systematic biopsy is indicated subsequently a prostate MRI is performed to indicate a combined targeted biopsy, **e** multivariate risk prediction tools incorporating prostate MRI for biopsy-decision management (systematic, targeted, or both), and **f** multivariate risk prediction tools (step 1) may indicate systematic biopsy and subsequently a prostate MRI is performed. Based on the outcome parameters of prostate MRI incorporated in MRI-multivariate risk prediction tools, these tools may navigate into further biopsy testing or into deferring biopsy testing. *PCa* prostate cancer, *PI*-*RADS* MRI suspicion score, *TBx* MRI-targeted biopsy, *SBx* systematic biopsy, *AS* active surveillance

It is important to develop and employ individualized risk-adapted strategies based on reliable risk prediction tools. Increasingly, the diagnostic work-up of prostate cancer detection is shifting towards using multivariate decision support tools that facilitate risk-adapted approaches [1]. Decision-making based on multivariate risk prediction tools can overcome the limitations of PSA-based screening (Fig. 1a). Furthermore, when multivariate risk prediction tools (Fig. 1b) and mpMRI (Fig. 1c) are utilized in the diagnostic work-up, several combinations become possible each of which clarifies underlying risk, indicating the need for and type of biopsy that maximizes benefits and minimizes harms (Fig. 1d–f). In this paper, we discuss each strategy evaluating diagnostic benefits and harms as defined above.

### Risk assessments based on PSA values only

The fact that PSA is a well-developed, easy to implement, and a cheap test makes PSA the mainstay in the decision for further clinical work-up. At opportunistic screening, informed men who request an early diagnosis should be given a PSA test and undergo a digital rectal examination (Fig. 1a) [19].

The occurrence of Gleason score ≥ 3 + 4 (or ISUP grading ≥ 2) prostate cancer even at low PSA levels precludes the setting up of an optimal PSA threshold for detecting non-palpable but clinically significant cancers [20]. Any PSA threshold involves a trade-off between sensitivity and specificity. Lowering the PSA cut-off value improves test sensitivity, but reduces specificity, leading to more false-positive tests and unnecessary interventions adding to harms. As a result of these limitations, using PSA values alone has fallen out of favor, leading to the introduction of multivariate risk prediction tools into clinical practice [1].

### Risk assessment based on multivariate risk prediction tools

Biopsy indications based on PSA cut-off values can be modified using clinical variables such as the initial PSA, PSA velocity, free/total PSA ratio, other serum kallikreins, prostate volume, and other predictors such as age, family history, and race alone or in combination within multivariate risk prediction tools [21]. Furthermore, urine markers (i.e., PCA3 and SelectMDx), and even genomic analyses could aid in risk stratification for biopsy indication.

Multivariate risk prediction models for prostate cancer diagnosis focus on correctly diagnosing patients with clinically significant prostate cancer, providing prognostic estimates thus aiding in clinical decision-making.

#### Performance measures: discrimination, calibration, and net benefit

Discrimination (i.e., separating people with disease from without disease), calibration (i.e., agreement between observed outcome and predicted risk), and net benefit (i.e., true positives minus false positives) are important indicators for evaluating the performance of risk prediction tools [22].

Accurate predictions discriminate between those with and those without clinically significant prostate cancer. Good discrimination means that men with significant cancer will consistently have higher predicted risks than those men without significant disease. Discrimination is important in diagnostic settings, because we want to separate men with and without clinically significant prostate cancer. To indicate the discriminative ability of risk prediction models for a binary outcome, the area under the Receiver-Operating Characteristic (ROC) curve is commonly used, which plots the sensitivity (true positive rate) against 1—(false-positive rate) for consecutive cut-offs for the probability of significant disease. In general, discrimination is not dependent on disease prevalence. Evaluation of prediction models should not rely solely on ROC curves, but should assess both discrimination and calibration.

Calibration refers to the agreement between observed outcomes and predictions. Calibration is more important in prognostic settings, because we would like to more precisely predict the risk of clinically significant prostate cancer. Calibration concerns itself directly with the estimated probabilities or predictive values. The positive predictive value is defined as the probability of clinically significant disease given a positive test result, and the negative predictive value is the probability of no significant disease given a negative test result. When a risk score is used, the continuous analog is the probability of disease given the value or range of the score. An assessment of calibration directly compares the observed and predicted probabilities, for which the disease prevalence is very important.

Some models over-/under-estimate the actual risk (have poor calibration), but can still separate those with disease and without disease (good discrimination). The reverse can also happen where a model has good calibration, but cannot discriminate between those with and without disease. Ideally, clinically useful models should be well calibrated and with high discrimination.

Net Benefit is a commonly used metric that weights the relative consequences in terms of the risk threshold at which a urologist would advise for further invasive diagnostic testing or treatment. Net benefit combines the number of true positives and false positives into a single “net” number. Conceptually, net benefit in business would be analogous to income minus expenditure [23]. In prostate cancer, true positives are clinically significant prostate cancer found; false positives are unnecessary prostate biopsies performed (hence ‘benefit-to-harm’ ratio). In prostate cancer diagnosis, we weigh the benefit of correct diagnosis strongly over the harms of negative biopsies.

The “exchange rate” reflects how many biopsies are acceptable to find one significant cancer [24]. In urological practice, there is no general agreement on this exchange rate or risk threshold for biopsy, so it is important to evaluate net benefit over a range of reasonable exchange rates or risk thresholds to advise men at risk and their urologists.

#### Risk thresholds

Risk-calculator outputs are on a sliding scale, indicating the likelihood of any cancer and clinically significant cancers (the definition of the latter often depends on the risk calculator). Often times, this continuum of estimated risks is categorized into low (not elevated), intermediate, and high (requiring biopsy) [25], thus helping to clarify an individual’s potential risk for clinically significant cancer, and thereby enabling the identification of only those men needing further testing, and contrarily identifying men at non-elevated risk and so reducing the number of unnecessary biopsies (Fig. 1b). Threshold values for biopsy, however, vary between risk calculators [12].

#### Risk calculators

There are several risk calculators designed for clinical use [25–32]. The European Randomized Study of Screening for Prostate Cancer-based Risk Calculator (RPCRC) [25] is applicable to Northern European populations and the North American Prostate Biopsy Collaborative Group (PBCG) Risk Calculator [31] is applicable to a more diverse radial group. Only four calculators are able to separately predict clinically significant prostate cancer (RPCRC [25], Prostate Cancer Prevention Trial (PCPT) 2.0 with or without (±) freePSA [30], and Sunnybrook RC [32]). Of these, only the RPCRC and PCPT 2.0 (± freePSA) have been externally validated in more than five studies [12].

#### Prediction of clinically significant prostate cancer

Four risk calculators have been shown to be able to predict the presence of clinically significant prostate cancer using biopsies, with areas under the curve (AUC; discrimination measure) values ranging from 0.71 to 0.77 in head-to-head comparisons, using patient data from a multicenter European and Australian population [21]. The RPCRC showed the highest discrimination [AUC 0.77 (95% CI: 0.73, 0.80)] indicating its benefit for daily practice [21]. Adjusting the calibration to prevalence improves the value of incorporating multivariable risk prediction tools in clinical decision-making. These results confirmed earlier analyses on the use of multivariable prediction tools [12] for prostate cancer diagnosis. Taken together, these results support the clinical use of multivariable risk prediction tools for the diagnostic work-up of men with suspected clinically significant prostate cancer. Although some risk calculators have greater efficiency than others, specific recommendations on which calculator to use is not stated within guidelines [1, 3]. Therefore, choice on which one to use depends on the mix of the local population and the clinical parameters available for data input.

## Prostate assessment by MRI

### From five-point Likert scale to binary MRI decision model

The PI-RADS (Prostate Imaging-Reporting and Data System) method for evaluating prostate MRI is designed to be used for the evaluation of patients at risk of having clinically significant prostate cancer, to decide who requires biopsy [33]. PI-RADSv2 assessments uses a five-point category scale indicating the likelihood (probability) that a combination of predefined MRI findings correlates with the presence of a clinically significant cancer for each identified lesion in the prostate gland [34]. A highly desirable quality of MRI is its high negative predictive value for clinically significant cancer [35, 36], meaning that men with negative tests may not need biopsies (Fig. 1c) [4, 5]. In practice, a binary MRI decision model for determining biopsy need has been introduced, with MRI-negative tests defined as PI-RADS assessment category 1 or 2, and MRI-positive tests defined as PI-RADS assessment categories 3, 4, or 5. For detecting clinically significant prostate cancer, this binary MRI decision model has been shown to have high sensitivity (0.91; 95% CI: 0.83, 0.95) and low specificity (0.37; 95% CI: 0.29, 0.46), when referenced to template-guided mapping verification biopsies [6]. False positives have been shown to occur predominantly within PI-RADS category 3 and 4 lesions, and less so in category 5 lesions [37, 38].

### From binary MRI decision model to four-point MRI-based risk assessment

Detection rates for clinically significant prostate cancer according to PI-RADS assessment categories vary significantly within and between patient cohorts (biopsy-naïve, prior negative biopsy, and prior positive biopsy in active surveillance) [37] and between studies [38]. The yield of clinically significant cancers per likelihood category depends on multiple factors, including histologic definitions employed (with higher yields for definitions that incorporate both tumor volume and tumor grade), and with the use of combined systematic biopsy cores with MRI-directed biopsy cores. Nevertheless, all published data consistently showed higher yields of clinically significant cancer with higher PI-RADS categories. For PI-RADS categories 3, 4, and 5 in biopsy-naïve men, the estimated detection rates of ISUP grade ≥ 2 (21%, 39%, and 73%) and ISUP grade ≥ 3 (6%, 16%, and 43%) showed stepwise higher yields [37]. The ISUP grade 1 (18%, 23%, and 19%) appears to decline over the PI-RADS categories 3, 4, and 5 [37].

It is possible to change the five MRI categories to a four-point category scale of risk assessment; viz low risk (PI-RADS 1 or 2), intermediate risk (PI-RADS 3), high risk (PI-RADS 4), and very high risk (PI-RADS 5). For these MRI risk categories, it is possible to ascribe different actions on the need for and type of biopsy such as those recommended by the EAU 2019 prostate cancer guidelines and the PI-RADS steering committee pathway white paper [1, 39].

### MRI performance and disease prevalence

Because PSA is widely used for indicating the need for biopsy, we should acknowledge that literature documented MRI utilities are only applicable to disease prevalences applicable to men whose risk was deemed to be sufficiently high to warrant an MRI before biopsy. The recent systematic analysis of Moldovan et al. indicated that in 48 studies (with 9613 patients), who underwent MRI, the median prevalence for any cancer was 50% [interquartile range (IQR), 36–58%] and was 33% (IQR, 28–37%) for significant cancer [36]. In the Cochrane analysis, focusing on biopsy-naïve men (with 5219 in 20 studies), the prevalence of any cancer was 53% (95% CI: 49–58%) and was 28% (95% CI: 24–33%) for significant cancer [6].

Disease prevalence impacts the clinical utility of prostate MRI. Take for example the PAIREDCAP study [40] which had a very high prevalence of ISUP grade ≥ 2 cancers (61%) in biopsy-naïve men detected by a combined approach of MRI-targeted and systematic biopsies. This overall 61% (182/300 men) prevalence resulted in a 70% (174/248 men) ISUP grade ≥ 2 detection rate in men with a positive MRI scan, with a marginal non-significant added benefit of targeted biopsies; the detection rates for targeted biopsy and systematic biopsy were 62% (154/248) and 60% (149/248) (*P *= 0.70), respectively. In comparison, the pooled prevalence in biopsy-naïve men was 28% in the Cochrane meta-analysis [6]. This 28% (95% CI: 24–33%) prevalence resulted in a 44% (95% CI: 39–50%) ISUP grade ≥ 2 detection rate in men with a positive MRI scan with a significant added benefit of targeted biopsies; the detection rates for targeted biopsy and systematic biopsy were 39% (95% CI: 33–46%) and 34% (95% CI: 28–41%) (*P *= 0.03), respectively. Therefore, when there is a very high risk of clinically significant prostate cancer, the benefit of a positive MRI decreases in comparison to a lower but elevated risk.

In MRI-negative men, the detection of ISUP grade ≥ 2 cancers by systematic biopsy was 15% (8/52 men), which was also almost double in comparison to 8% (95%CI: 6–12%) in the Cochrane review. As a result, in the Cochrane review, 12–13 MRI-negative men need to be biopsied to detect one man with ISUP grade ≥ 2 cancer, which maybe consider as an unfavorable exchange rate, whereas for the PAIREDCAP study population, this number is 6–7 men, which is more acceptable. Therefore, when there is an elevated risk of clinically significant prostate cancer, a negative MRI should not be used to avoid biopsy (Fig. 1d) because of resulting increases in the rate of missed significant prostate cancer [36].

Similarly, the utility of MRI at lower risk profiles such as in population screening also cannot be drawn from the published data. In fact, we need to be aware that in men with lower risk profiles, the false-positive rate of MRI will increase [41], and the low specificity of MRI will result in increased numbers of false positives. This could unnecessary increase biopsy rates impacting adversely on the benefit-to-harms ratio.

We, therefore, need to create new risk categories that consider both the likely prevalence of clinically significant prostate cancer and the results of MRI scans, when choosing which men need biopsy and who can be safely monitored. These revised MRI integrated risk categories can then be used to also indicate biopsy method, route, and number of cores, to maximize benefits and reduce harms. These will differ by geographic population, by institutional preferences, and between patient cohorts (first biopsy, repeat biopsy, and active surveillance).

## Integrating multivariate risk prediction tools with MRI

There are multiple ways in which multivariate risk prediction tools can be combined with MRI results to improve prostate cancer diagnosis. These include (1) using prediction tools to indicate the need for an MRI, (2) to indicate the need for biopsy particularly for equivocal cases after MRI results, and (3) to indicate the extent of and the approaches for tissue sampling.

### Indicating need for MRI

Multivariate prediction tools can be used to indicate the need for an MRI (Fig. 1d). The purpose of upfront MRI is twofold: (1) to mitigate the unrestraint use of MRI testing [14], and (2) to enrich the population prevalence to a high enough level, where MRI has been shown to have clinical utility as discussed above. When multivariate risk prediction tools indicate that risk is not high enough to perform a biopsy or MRI, avoiding MRI will result in limiting the false-positive outcomes of MRI testing [42], and, therefore, reduce the number of biopsies undertaken. This means that some clinically significant cancers will not be immediately diagnosed (just as in the pre-MRI era but with lesser frequency), requiring robust follow-up regimens to catch emerging over time.

When multivariate risk prediction tools indicate the need for systematic biopsy, an MRI should be undertaken in men likely to benefit most from a PI-RADS compliant multi-parametric MRI scan. This typically includes asymptomatic men with PSA levels between 2 and 10 ng/ml [1]. A comprehensive scanning approach may not be needed for symptomatic men with a very high risk of prostate cancer based on positive digital rectal examination, very high PSA levels, and multivariable risk-calculator estimates [10]; for these men, a more limited approach without contrast medium may be sufficient.

### Indicating need for biopsy

**Fig. 2:**
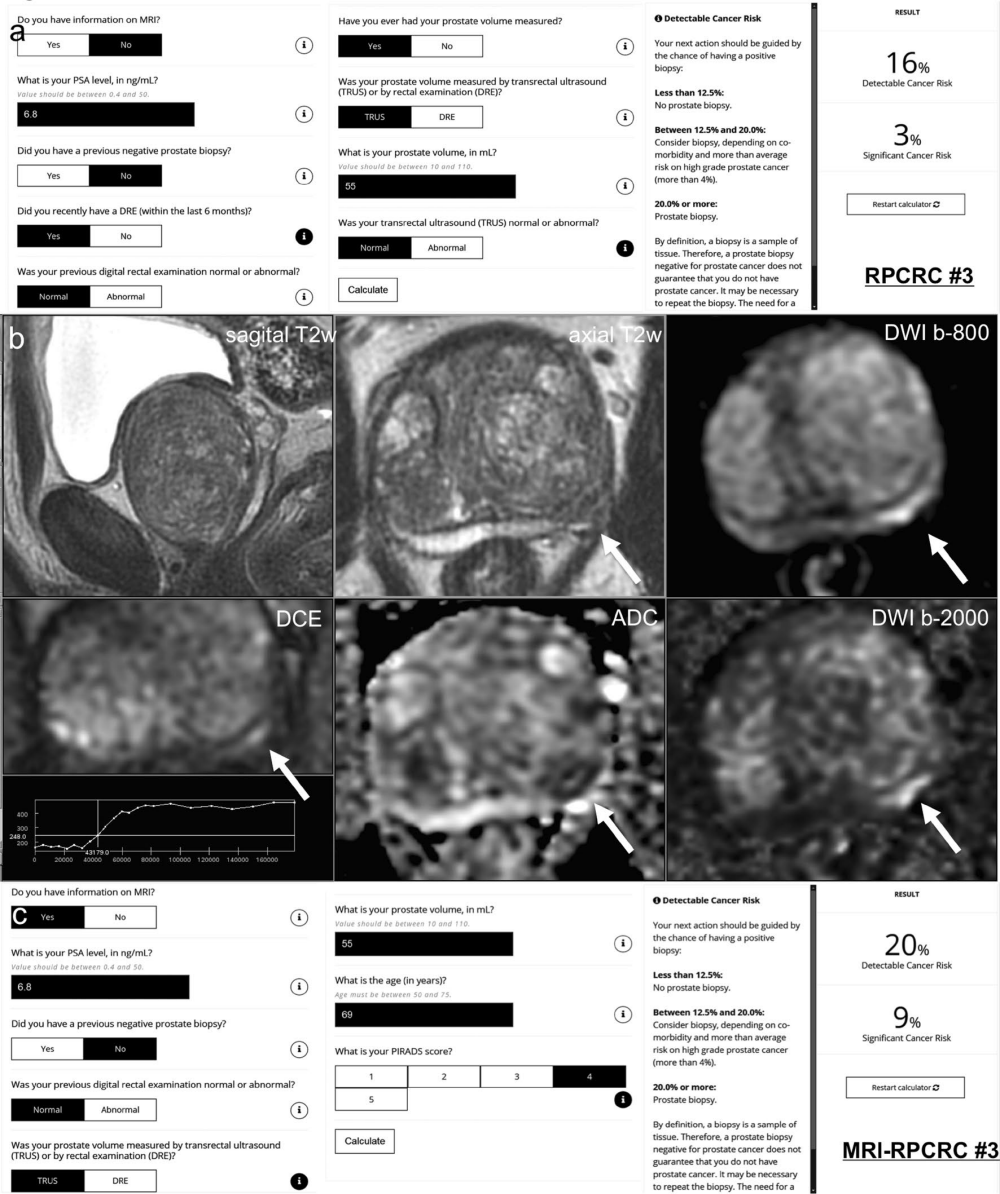
Case example: a 69-year-old man with a PSA of 6.8 ng/ml. Digital rectal examination (DRE) was normal, and transrectal ultrasound (TRUS) showed a prostate volume of 55 ml, without any hypo-echoic lesions found on ultrasound. The PSA density was 0.12 ng/ml^2^ (6.8/55). *Does this biopsy-naive man deserve an MRI and/or biopsy?* (**1**) In this biopsy-naïve man, the chance of finding any prostate cancer with further testing is 32%, based on the *ERSPC-RC#2* (including PSA only in biopsy-naïve men) [49], suggesting a systematic biopsy is justified. (**2**) The chance of finding any prostate cancer and clinically significant prostate cancer with further testing is 16% and 3%, respectively, based on the *ERSPC-RC#3* (including PSA, DRE, TRUS, and TRUS volume) [49] (**a**). Including DRE, TRUS, and TRUS volume in the risk calculation gives a lower risk estimation of having a biopsy-detectable clinically significant prostate cancer. The next action should be guided by the chance of having a positive biopsy: (1) less than 12.5% → no prostate biopsy (and no MRI); (2) between 12.5 and 20.0% → consider biopsy (and MRI), depending on co-morbidity and more than average risk on high grade prostate cancer (more than 4%); (3) 20.0% or more → prostate biopsy (and MRI). In this man, a systematic biopsy is justified, based on the ERSPC-RC#3. (**3**) A 3T multi-parametric MRI was performed (**b**), showing a large prostate gland with compression of the peripheral zone, and a suspected lesion for clinically significant prostate cancer at the left peripheral zone dorsolateral, at the mid-prostate, 12 × 4 mm, without extraprostatic extension or seminal vesicle invasion; PI-RADS assessment score 4 (4/4/+), based on low signal intensity on T2w-imaging (4), high signal intensity on DWI (b-800 and b-2000 (calculated) combined with low signal intensity on ADC (4), and focal enhancement (+). Based on high yields of clinically significant prostate cancer (> 30–40%) in an MRI lesion with a PI-RADS assessment score 4 (Sects. 3.2 and 4.2) [37], a targeted biopsy is indicated. (**4**) The chance of finding any prostate cancer and clinically significant prostate cancer with further testing is 20% and 9%, respectively, based on the *MRI-ERSPC-RC#3* (including PSA, DRE, TRUS, TRUS volume, and MRI PI-RADS score) [49] (**c**). In this man, a systematic and targeted biopsy is justified. *Biopsy considerations* the morphology of the suspected peripheral zone lesion is long and small; targeted cores of the lesions combined with targeted cores from the penumbra (focal saturation) could be sufficient. *Biopsy protocol and pathology findings* Protocolled 12 systematic biopsies and 2 targeted biopsies were performed under MRI/US fusion guidance. 12 systematic biopsies showed benign prostatic tissue only. 2 targeted biopsy cores showed Gleason score 3 + 4 (ISUP group 2) with 3 mm and 4 mm maximum cancer core length, without cribriform or intraductal growth pattern

**Fig. 3:**
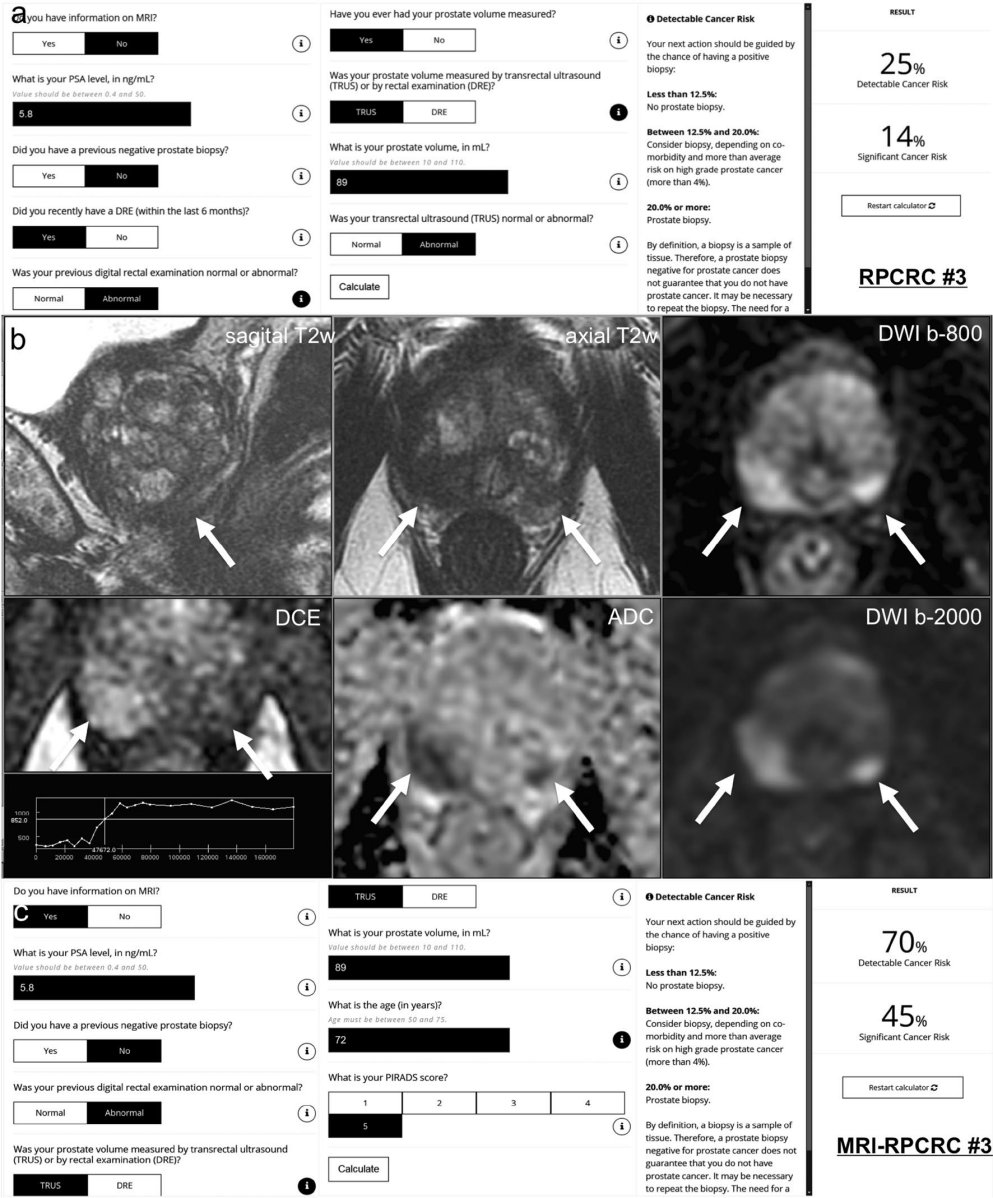
Case example. a 72-year-old man has a PSA of 5.8 ng/ml with lower urinary tract symptoms. Digital rectal examination (DRE) showed a right-sided abnormal prostate, classified as cT2 tumor. Transrectal ultrasound (TRUS) showed a prostate volume of 89 ml, with a right-sided hypo-echoic lesion found on ultrasound. The PSA density was 0.07 ng/ml^2^ (5.8/89). *Does this biopsy-naive man deserve an MRI/biopsy?* (**1**) In this biopsy-naïve man, the chance of finding any prostate cancer with further testing is 29%, based on the *ERSPC-RC#2* (including PSA only in biopsy-naïve men) [49], suggesting a systematic biopsy is justified. (**2**) The chance of finding any prostate cancer and clinically significant prostate cancer with further testing is 25% and 14%, respectively, based on the *ERSPC-RC#3* (including PSA, DRE, TRUS, and TRUS volume) [49] (**a**). In this man, a systematic biopsy is strongly recommended, based on the ERSPC-RC#3. (**3**) A 3T multi-parametric MRI was performed (**b**), showing a large prostate with compression of the peripheral zone, and a suspected lesion for clinically significant prostate cancer at the right peripheral zone dorsolateral, at the apex, 17 × 15 mm, without extraprostatic extension or seminal vesicle invasion; PI-RADS assessment score 5 (5/5/+), based on low signal intensity on T2w-imaging (5), high signal intensity on DWI (b-800 and b-2000 (synthetic/calculated) combined with low signal intensity on ADC (5), and focal enhancement (+). Based on high yields of clinically significant prostate cancer (> 70%) in an MRI lesion with a PI-RADS assessment score 5 (Sect. 3.2 and 4.2) [37], a targeted biopsy is strongly recommended in this biopsy-naïve man. A separate suspected lesion in the dorsal left peripheral zone in the apex categorized as PI-RADS score 4 (4/4/−) suggests multifocal prostate cancer. (**4**) The chance of finding any prostate cancer and clinically significant prostate cancer with further testing is 70% and 45%, respectively, based on the *MRI-ERSPC-RC#3*, including PSA, DRE, TRUS, TRUS volume, and MRI PI-RADS score [49] (**c**). In this man, a systematic and targeted biopsy is advised. *Biopsy considerations* The morphology of the suspected peripheral zone lesion in the right apex is large; targeted cores of the lesion will most likely hit the lesion. To limit undersampling, up to five cores might be sufficient [50]. The morphology of the suspected peripheral zone lesion in the left apex is small. Although targeted cores of the lesion most likely will hit the lesion, still, there is reasonable chance of undersampling or missing the lesion. A focal saturation biopsy might be most accurate [39]. *Biopsy protocol and pathology findings* Protocolled 12 systematic biopsies and 2 targeted biopsies in each suspected lesion were performed under MRI/US fusion guidance. Right-sided 6 systematic biopsies showed in 3 cores Gleason score 3 + 4 (ISUP group 2) with 1 mm, 10 mm, and 3 mm maximum cancer core length. Right-sided 2 targeted biopsies showed in 2 cores Gleason score 3 + 4 (ISUP group 2) with 14 mm and 7 mm maximum cancer core length, without cribriform or intraductal growth pattern. Left-sided 6 systematic biopsies showed benign prostatic tissue. Left-sided 2 targeted biopsies showed in 2 cores Gleason score 3 + 3 (ISUP group 1) with 6 mm and 1 mm maximum cancer core length, without cribriform or intraductal growth pattern

Magnetic resonance imaging results can help to refine risk stratification (by providing a more accurate prostate volume compared to clinical estimates and by the PI-RADS suspicion category) (Figs. 2, 3). If the risk remains high despite a negative MRI result, MRI results will not affect the number of men undergoing systematic biopsies. Thus, there will be little impact on over-diagnosis rates in men with negative scans [40].

The prevalence of clinically significant cancer in intermediate PI-RADS category 3 patients varies from one in five (21%) to one in six (16%), depending on previous biopsy status [37]. Although this PI-RADS 3 category significant prostate cancer prevalence is low in comparison to PI-RADS category 4 (range 33–39%) and 5 lesions (range 60–73%) [37], the proportion of men with significant disease is not inconsequential. As a result, in some clinical practices, the decision to biopsy PI-RADS category 3 men is influenced by a range of clinical factors including PSA kinetics, PSA density, previous biopsy results, and patient preferences [43, 44]. This argues for including multivariate prediction tools for biopsy decision-making in men with intermediate PI-RADS 3 category lesions.

For high-risk men, positive MRI results can aid in the choice of biopsy methods (targeted with or without systematic biopsy), to maximize diagnostic yields of significant cancers [13, 14] as discussed in the next section.

### Indicating biopsy extent and approaches

There is robust debate about how to best use MRI results; to increase yields of significant cancers versus reducing over-diagnoses of insignificant cancers. Magnetic resonance imaging results can be used in multiple ways [45]. (1) The ‘combined biopsy pathway’ where patients with negative MRI undergo scheduled systematic biopsy and men with positive MRI undergo both systematic and MRI-directed biopsy maximizes the diagnostic yields of significant cancers [8, 9]. (2) The ‘MRI-pathway’ is distinct in that men with negative scans are not biopsied at all, and men with positive scans undergo only MRI-directed biopsies (without systematic cores). The advantage of the MRI-pathway is to reduce the number of men needing biopsies and to reduce the total number of biopsy cores taken, thus helping to reduce over-diagnoses of insignificant disease. A mixed biopsy approach can also be used.

The combined biopsy pathway uses the intrinsic MRI localization information to influence biopsy approaches. The location and size of MRI lesions can indicate the preference for limited MRI-directed biopsy cores only, or the need for additional focal saturation biopsy cores, or the additional need for systematic biopsy cores (Fig. 1e) as per the recommendations of the 2019 EAU guidelines and the PI-RADS Committee Pathway white paper [1, 39].

For example, large PI-RADS category 5 lesions might be biopsied by a limited number of targeted biopsy cores, omitting systematic biopsies. In men with PI-RADS category 3 or small category 4 prostate lesions, the combined use of elevated risk-calculator findings and MRI location information may indicate the need for using MRI-targeted and systematic biopsy approaches to gain maximal diagnostic yields. When over-diagnosis is a concern, for example in biopsy-naïve men with PI-RADS category 3 or small category 4 prostate lesions, biopsies using MRI-targeted and focal saturation biopsy approaches may be sufficient [39]. In men with PI-RADS category 3, lesions with low clinical suspicion on risk calculator or on PSA density will likely not be biopsied at all [44]. On the other hand, men with non suspicious MRI who are deemed to be at high risk using clinical and biochemical parameters would need to be biopsied in a systematic manner, even in the absence of definable MRI targets. Those men at low-risk using clinical and biochemical parameters and negative MRI scans can safely avoid biopsy and could be discharged from urological care.

**Fig. 4:**
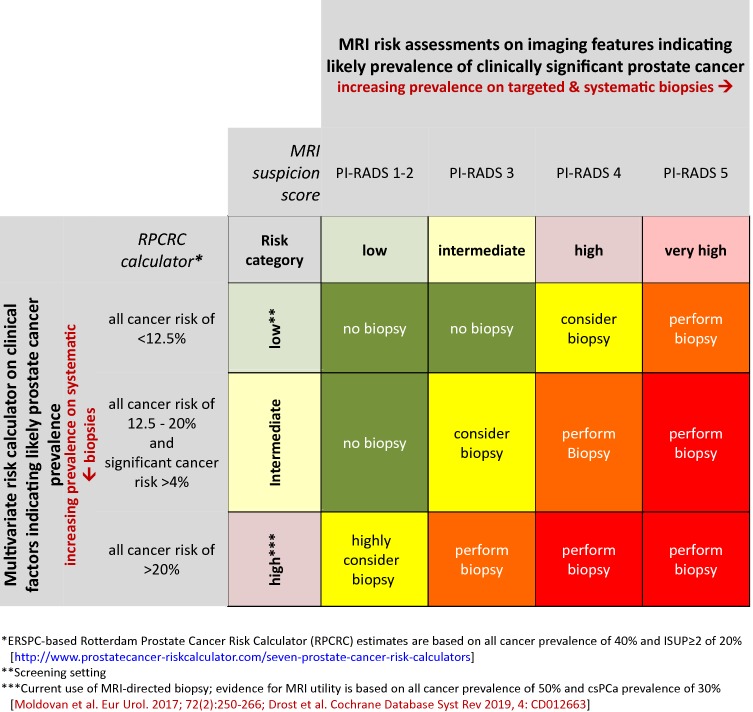
Matrix table based on the validated ERSPC-based Rotterdam prostate cancer risk calculator (RPCRC) and on MRI risk assessments. The continuum of estimated risks of having a biopsy-detectable prostate cancer in the RPCRC is categorized into low (no elevated risk; less then 12.5%), intermediate (elevated risk; between 12.5 and 20%), and high (elevated risk; 20% or more). Men with high risk of having a biopsy-detectable prostate cancer require biopsy. Also men with intermediate risk combined with a more than average risk on clinically significant prostate cancer (more than 4%) require biopsy [25]. Clinically significant prostate cancer in RPCRC is defined as any Gleason ≥ 4 grade, or primary and secondary Gleason ≤ 3 with ≥ 50% positive cores or total cancer core length of ≥ 20 mm [25]. The MRI risk assessment is categorized into low (PI-RADS 1 or 2), intermediate (PI-RADS 3), high (PI-RADS 4), and very high risk (PI-RADS 5) of having a biopsy-detectable clinically significant prostate cancer. Each cell ascribes a different biopsy action. This matrix table bridges the gap to new thresholds of developing and not-yet validated MRI prediction tools and may guide biopsy-decision management on individual patients in the increasingly complex, multivariate approach of prostate cancer diagnosis

In each of the above clinical scenarios, underlying risk and imaging findings are subjectively combined for deciding on biopsy need and approach. Given that it is possible to predict underlying risk using multivariate prediction tools and MRI assessments, a combined more objective approach becomes possible (Fig. 4) to help decide on biopsy actions for combined risk profiles as discussed in the next section.

### Combining multivariable risk prediction tools including MRI

Multivariate risk prediction tools that include MRI suspicion scores also have the potential to substantially lower the number of biopsies and the detection of clinically insignificant prostate cancer, at a low price of missing some clinically significant prostate cancer. The trade-off between significant cancer detected and missed, in combination with the avoided unnecessary biopsies (net benefit), depends on the chosen risk thresholds for biopsy (exchange rates) as discussed above. In the current context, this is the probability threshold used to determine whether a patient is classified as being positive or negative for significant prostate cancer and, therefore, needs biopsy [23]. In biopsy-naïve men, higher net benefits are obtained at risk thresholds above 10% in MRI-multivariate risk prediction models [42]. That is, in a man with an MRI, adjusted multivariate risk calculator of > 10% likelihood of cancer is more likely to benefit from biopsy than not (Fig. 1e).

When multivariate risk prediction models are used, the calculated risk is a continuum. For clinical practicality, thresholds for performing biopsy need to be defined for multivariate risk prediction tools that include MRI results [15, 16, 46–48]. Since multivariate risk prediction models incorporating MRI results have not yet been validated, these model thresholds are not yet available in the literature.

To bridge this knowledge gap, a matrix table is, therefore, proposed to categorize men with different risk profiles, based on validated multivariate risk prediction tools and on MRI risk assessments (Fig. 4). The continuum of estimated risks in multivariate risk prediction tools is categorized into low (not elevated), intermediate, and high (requiring biopsy). The MRI risk assessment is categorized into low (PI-RADS 1 or 2), intermediate (PI-RADS 3), high (PI-RADS 4), and very high (PI-RADS 5). Each cell within the matrix has an ascribed biopsy action, taken from the recommendation of the EAU 2019 prostate cancer guidelines and the PI-RADS steering committee pathway white paper [1, 39]. This proposal may guide biopsy-decision management on an individual basis in the increasingly complex approach of prostate cancer diagnosis as illustrated in Figs. 2 and 3.

Clearly, multivariate risk prediction tools will need to be adjusted when the use purpose is changed. As already mentioned, adjustments to thresholds will likely be needed for deciding on biopsy strategies after MRI to optimize net benefits (Fig. 1e). However, adjustment will be different when using them for deciding on the need for MRI (Fig. 1d), for decision to biopsy and biopsy type (e.g., focal saturation biopsy cores, or the additional systematic biopsy) (Fig. 1e), or at both for the need of MRI and decision to biopsy and biopsy type (Fig. 1f). Threshold adjustments will also be required to address changing risk-over-time. For example, in active surveillance if the initial low risk or intermediate risk is stable over time on multivariate analysis, the indication to further (biopsy) testing may be deferred. The advantages and disadvantages of three variations of mpMRI in combination with multivariate risk prediction tools discussed above (Fig. 1d–f) are summarized in Table 1.

**Table 1:** Utility of multivariate risk prediction tools, with and without MRI, in the prostate cancer diagnostic work-up

Risk stratification by	Stratifying men to indicate the need for:	Stratified risk (test result)		Action for further testing	Purpose of action	(Dis)advantages
Multivariate risk prediction tool not including MRI (Fig. 1d)	Biopsy and MRI	High	Yes Yes Yes	MRI Targeted biopsy Systematic biopsy	Maximizing diagnostic yield	Pros 1. Owing to increased pre-test probability, the post-test probability (PPV) of both MRI and biopsies will increase 2. When stratified to low-risk, both diagnostic tests (MRI and biopsy) can be omitted. Resulting in reductions of low-risk PCa detection Cons 1. Does not distinguish between the indication for biopsy or for MRI 2. No discrimination of added value for MRI or biopsy can result in too much testing 3. Missed clinically significant prostate cancers in non-biopsy patients
		Intermediate	Yes Yes Yes	MRI Targeted biopsy Systematic biopsy	Maximizing diagnostic yield	
		Low	No No No	MRI Targeted biopsy Systematic biopsy	Reducing MRI and biopsies Reducing targeted biopsies Reducing systematic biopsies Reducing low-risk PCa detection	
Multivariate risk prediction tool including MRI (Fig. 1e)	Biopsy	High	Yes No	Targeted biopsy Systematic biopsy	Optimizing diagnostic yield Reducing systematic biopsies	Pros 1. Optimal pre-biopsy information gathered for biopsy indication. 2. Large PI-RADS category 5 lesions will be categorized in the ‘high-risk’ group, and will probably be biopsied in targeted approach only, omitting systematic biopsies 3. The added value of combining MRI within a multivariate prediction tool is mostly focussed on reducing the high false-positivity of MRI (low specificity and low PPV). Probably PI-RADS category 3 and 4 lesions will fall in this ‘intermediate’ category. With the combination of elevated risk, based on clinical parameters, these lesions will be biopsied in a targeted approach but also systematic biopsies will be performed to gain maximal diagnostic yield 4. Negative MRI with a high-risk profile on clinical parameters could be biopsied in a systematic manner 5. Low-risk profile (negative MRI and low-risk on clinical parameters) may save further testing Cons 1. No reduction of prostate MRI
		Intermediate	Yes Yes	Targeted biopsy Systematic biopsy	Maximizing diagnostic yield	
		Low	No No	Targeted biopsy Systematic biopsy	Reducing targeted biopsies Reducing systematic biopsies Reducing low-risk PCa detection	
Multivariate risk prediction tool (step 1) not including MRI (Fig. 1f)	MRI	High	Yes	MRI	Maximizing diagnostic yield	Pros 1. Distinguish between indication of biopsy and MRI. The first multivariate prediction tool (step 1) stratifies who should get an MRI. This may lead to reduction of MRI (and subsequent reduction of biopsies in the low-risk group. Men in the high-risk group will get an MRI 2. The results from this MRI will be combined in a second multivariate prediction tool (step 2) after MRI, and may further lead to further risk stratification for the indication who to biopsy, and in which approaches to use 3. Men with a negative MRI and with a high-risk profile on clinical parameters could be categorized in the intermediate or low-risk group, depending on the thresholds that will be chosen and if biopsy is required, a systematic approach could be used 4. Thresholds in step 1 might differ from step 2 Cons 1. Missing significant cancers as the trade-off for the reduction of MRI scans done
		Intermediate	Yes	MRI	Maximizing diagnostic yield	
		Low	No No No	MRI Targeted biopsy Systematic biopsy	Reducing MRI Reducing targeted biopsies Reducing systematic biopsies Reducing low-risk PCa detection	
Multivariate risk prediction tool including MRI (step 2) (Fig. 1f)	Biopsy	High	Yes No	Targeted biopsy Systematic biopsy	Optimizing diagnostic yield Reducing systematic biopsies	
		Intermediate	Yes Yes	Targeted biopsy Systematic biopsy	Maximizing diagnostic yield	
		Low	No	Systematic biopsy if needed	Reducing targeted biopsies Reducing systematic biopsies Reducing low-risk PCa detection	

## Conclusion

Multivariate risk prediction tools used before MRI support physicians and patients deciding on the need for MRI before a biopsy consideration. The major benefit of pre-biopsy MRI in the diagnostic work-up is to promote individualized risk-adapted approaches for biopsy-decision management. The current benefits of pre-biopsy MRI for prostate cancer diagnostic work-up presuppose a high prevalence of clinically significant cancer. Multivariate risk prediction tools in which MRI results are incorporated can support physicians and patients in biopsy decision-making in appropriately chosen patients. Several clinical scenarios incorporating MRI are conceivable. Each diagnostic approach has net-benefit trade-offs between benefits and harms, based on improved diagnostic yields and reduced biopsy testing and reduced detection of indolent prostate cancer. Further data on the utility of multivariable risk prediction models that incorporate MRI information are awaited.
